# Correlation between phenotypic disc diffusion test and PCR molecular test for detection of methicillin resistance in Staphylococcus epidermidis and Staphylococcus hominis

**DOI:** 10.1099/jmm.0.002207

**Published:** 2026-11-03

**Authors:** Lorena Gonzalez-Lopez, Ruben Morelos-Ramirez, Roberto Cabrera

**Affiliations:** 1 Departamento de Salud Pública, Facultad de Medicina, Universidad Nacional Autónoma de México, Ciudad de México, Mexico

**Keywords:** cefoxitin, coagulase-negative staphylococci (CoNS), disc diffusion test, methicillin resistance, neonatal sepsis, oxacillin

## Abstract

**Introduction.** Coagulase-negative staphylococci (CoNS), particularly *Staphylococcus epidermidis*, are leading opportunistic pathogens in neonatal intensive care units, frequently associated with late-onset sepsis and medical device colonization.

**Gap statement.** Cefoxitin disk diffusion has been adopted internationally as a surrogate marker for *mecA*, but its accuracy in CoNS is heterogeneous; high resistance rates and erratic phenotypic expression in species like *Staphylococcus hominis* indicate that the existing phenotypic-only approach does not transfer reliably between species and cohorts.

**Aim.** Evaluate the diagnostic accuracy of oxacillin agar dilution (AD) and cefoxitin disc diffusion against *mecA* PCR in 664 coagulase-negative staphylococcal isolates collected over an 8-year period (2006–2014) at a tertiary-level hospital.

**Methodology.** Phenotypic detection of methicillin resistance was performed by oxacillin AD with 4% NaCl/24 h and by cefoxitin disk diffusion (≤24 mm), using *mecA* PCR as the reference standard; inducible clindamycin resistance (iMLS_B) was screened by D-test. All isolates were stratified by species (*S. epidermidis, S. hominis*) and cohort (neonatal versus adult), with concordance quantified using Cohen’s *κ* coefficient under the Landis and Koch framework.

**Results.** *S. epidermidis* accounted for 87.2% and *S. hominis* for 8.6% of isolates, with 83.1% originating from neonates. In neonatal *S. epidermidis*, *mecA* prevalence reached 80%. Oxacillin AD demonstrated superior performance with 87.1% sensitivity (neonates) and 78.3% (adults), with moderate agreement in both subgroups (*κ*=0.44–0.54, respectively). The D-test further revealed a 20.8% prevalence of the inducible clindamycin resistance phenotype (*iMLS_B*) in neonatal *S. hominis*, indicating a high risk of therapeutic failure despite an apparently susceptible routine antibiogram, a prevalence nearly 20-fold higher than that observed in neonatal *S. epidermidis* (1.0%, n=5), identifying *S. hominis* as a particularly problematic inducible-clindamycin-resistance reservoir in the NICU setting.

**Conclusion.** High resistance rates and erratic phenotypic expression in species like *S. hominis*, together with the unequal accuracy of phenotypic methods between neonates and adults, require a species-specific diagnostic approach. While oxacillin AD remains a robust predictor for both species and cohorts in this setting, systematic D-test implementation and *mecA* molecular confirmation are essential to ensure effective antimicrobial therapy.

**Impact Statement**

This study provides an update on the escalating antimicrobial resistance of coagulase-negative staphylococci within tertiary-level neonatal care. By comparing 664 clinical isolates from neonatal and adult populations, it is demonstrated that the Neonatal Intensive Care Unit acts as a high-pressure ecological niche, driving constitutive clindamycin resistance (cMLS_B) to exceed 70%, a stark contrast to the 43% observed in non-Intensive Care Unit adult settings. Furthermore, current diagnostic paradigms are challenged by these results, which reveal the superior reliability of oxacillin agar dilution compared with cefoxitin disc diffusion for *mecA* detection in neonatal *Staphylococcus epidermidis* isolates. The identification of a significant ‘silent risk’ in *Staphylococcus hominis –* where 20.8% of isolates exhibit inducible resistance (iMLS_B) – underscores the mandatory implementation of the D-test to prevent therapeutic failure. This research represents a significant step forward in neonatal infectiology by integrating clinical origins with molecular precision, providing essential evidence for refining empirical antibiotic protocols and hospital surveillance strategies to protect highly vulnerable preterm infants.

## Data Summary

The authors confirm all supporting data, code and protocols have been provided within the article or through supplementary data files.

## Introduction

The *Staphylococcus* genus is comprised of a diverse group of more than 50 species and subspecies of Gram-positive cocci. Based on the capacity for coagulase enzyme production, the genus is categorized into two main phenotypic groups, coagulase-positive staphylococci and coagulase-negative staphylococci (CoNS) [1, 2]. Initially, this concept was regarded primarily as a classification based on diagnostic procedures (e.g. serum clot formation or PCR using the coagulase gene *coa*); however, it subsequently evolved into a clinical diagnostic approach to differentiate between the pathogenic species *Staphylococcus aureus* and a group of staphylococci formerly classified as non-pathogenic [3].

CoNS represent a diverse group of opportunistic pathogens, among which six species are most frequently associated with human disease, namely *Staphylococcus epidermidis*, *Staphylococcus haemolyticus*, *Staphylococcus saprophyticus, Staphylococcus capitis, Staphylococcus hominis and Staphylococcus lugdunensis* [4, 5]. The pathogenic potential of CoNS is primarily related to certain virulence factors, especially biofilms, toxins and antimicrobial resistance [1, 5]. To determine whether CoNS is a true cause of infection rather than a contaminant, clinical and microbiological diagnostic criteria are applied. These include obtaining two or more positive isolates of the same species from separate longitudinal blood cultures (or distinct sterile sites), clinical symptom improvement following targeted therapy, the presence of an indwelling medical device, a time to positivity of less than 16 h and demonstrated resistance profiles [6]. Although the recovery of two or more isolates of the same CoNS species from separate longitudinal blood cultures or sterile sites is the operational threshold to apply before considering an isolate clinically significant, the presence of a different pathogen in the same specimen does not, by itself, exclude a contributory role for CoNS in true polymicrobial infections; however, a single CoNS isolate obtained in the presence of a primary pathogen is usually regarded as a likely contaminant, and the clinical context should guide interpretation [7].

To date, the most frequent CoNS species associated with human infections and the causative agent of nosocomial bacteraemia is *S. epidermidis,* due to its ability to form biofilms on the surface of implantable medical devices [8, 9]. For this reason, these bacteria are associated with the use of catheters, prostheses and various polymer and metal implants, which are widely used in the context of modern medicine [10, 11]. Additionally, the colonization of different parts of the skin and mucous membranes of patients by CoNS is a key source of endogenous infections, mainly in newborns, immunocompromised patients and critically ill patients [3, 12, 13]. Notably, CoNS are established as a primary cause of neonatal sepsis, significantly impacting morbidity and mortality, with prevalence ranging from 1.9 to 4.8% in the general neonatal population and reaching 9.4% in very low birth weight infants [14]. Some of the pathologies related to CoNS in this population are retinopathy of prematurity, necrotizing enterocolitis, white matter injury and bronchopulmonary dysplasia, which are mainly caused by *S. epidermidis* [14].

Antimicrobial resistance constitutes a significant public health burden concerning the management of bacterial infections, primarily associated with healthcare-associated infections (HAIs) [15, 16]. Methicillin was the first semisynthetic penicillin derivative antibiotic effective against *S. aureus* [17]. However, within 3 years of introduction, the first methicillin-resistant strains were isolated [18, 19]. Resistance to methicillin is due to the presence of mobile genetic elements, namely the staphylococcal cassette chromosome *mec* (SCC*mec*) that contains the *mecA* gene, responsible to produce an altered target penicillin-binding protein (PBP2a) [18, 20]. Treatment of CoNS is becoming increasingly complex due to the emergence of methicillin resistance (MR). In *S. epidermidis* MR has been detected in 75–90% of all hospital isolates carrying the staphylococcal SCC*mec* [12, 21]. MR can be detected by disc diffusion test with β-lactam molecules such as oxacillin or cefoxitin [7]. Particularly, cefoxitin has demonstrated itself to be a more sensitive and specific marker of *mecA*-mediated MR and it was considered the agent of choice for diffusion tests by the Clinical and Laboratory Standards Institute (CLSI), which superseded the former National Committee for Clinical Laboratory Standards [22].

A total of 664 CoNS isolates were obtained from patients with HAIs, predominantly neonates, over an 8-year period. All samples were obtained from the National Institute of Perinatology (INPer) ‘Dr. Isidro Espinoza de los Reyes’, that serves women with high-risk pregnancy. The primary objective involved species identification and the correlation of cefoxitin disc diffusion assay with PCR-based detection to characterize methicillin-resistant *S. epidermidis* isolates within this population.

## Methods

### Isolates

A total of 664 *Staphylococcus* spp. isolates from patients with HAIs were collected over an 8-year period (March 2006 to November 2014) at INPer ‘Dr. Isidro Espinoza de los Reyes’.

### Phenotypic identification and coagulase-negative staphylococci

Species identification of each of the 664 strains was carried out in a VITEK equipment (bioMérieux SA, 376 Chemin de l'Ome, France), following the manufacturer’s instructions. Upon arrival at the Bacterial Pathogenicity Laboratory of the Faculty of Medicine, Universidad Nacional Autónoma de México, each of the strains was cultured in Petri dishes, containing brain heart infusion agar (OXOID Ltd, Basingstoke, Hampshire, England) and mannitol salt agar (MA) (OXOID Ltd, Basingstoke, Hampshire, England). After incubation for 18–24 h at +37 °C in an Isotemp microbiological incubator 750D (Thermo Fisher Scientific, Waltham, Massachusetts, USA), colonies were examined and the morphology was verified after Gram staining (HYCEL de México S. A. de C. V. Av. Mexico City). The presumptively identified *S. epidermidis* and other CoNS colonies were examined for coagulase activity by tube plasma agglutination test (REMEL Coagulase Plasma, Santa Fe Drive, Lenexa, KS, USA). After characterization, the isolated strains were stored in an Ultima II ultra-low freezer, ULT-390–9 A31 (REVCO Technologies Asheville, NC, USA) at −80 °C in a 20% skim milk suspension, until use.

### DNA extraction and purification

Genomic DNA was isolated using the Lysostaphin-Lysozyme method of Hookey [23], only modifying the lysis step. Individual colonies were used to inoculate tryptic soy broth and grown overnight, after which genomic DNA was extracted. Briefly, bacterial cells were resuspended in 250 µl of lysis solution (lysozyme 50 mg ml^−1^; lysostaphin 1 mg ml^−1^ in 25 mM Tris/HCl, 10 mM EDTA; Sigma-Aldrich, St. Louis, MO, USA) and incubated at 37 °C for 90 min. The sample was then warmed to 95 °C for 10 min and centrifuged at 8,000 ***g***. The lysate was extracted with equal volumes of phenol/chloroform, and the DNA was precipitated from the aqueous phase with 2-propanol, resuspended in 100 µl of sterile distilled water and stored at 4 °C. The integrity of the DNA preparations was determined by gel electrophoresis (5 µl aliquots) and spectrophotometry at A260 nm.

### Duplex PCR for *coa and mecA* genes

Genomic DNA was extracted from all isolates, and a duplex PCR procedure was performed for the simultaneous detection of the *mecA* and *coa* genes, following the protocol described by Hookey *et al*. [23]. Identification of *S. aureus* was determined using the *coa* gene as a specific marker, whereas MR was genetically confirmed through the presence of the *mecA* gene. The primers utilized, as previously validated in our laboratory [24], included: for *coa,* F-5′-AACTTGAAAT-AAAACCACAAGG-3′ and R-5′*-*TACCTGTACCAGCATCTCTA-3′ (yielding a 177 bp amplicon); and for *mecA*, F-5*′*-ATGGCAAAGATATTCAACTAAC-3′ and R-5*′*-GAGTGCTACTCTAGCAAAGA-3′ (yielding a 458 bp amplicon).

PCR was performed on a Bio-Rad C1000 thermal cycler (Bio-Rad Laboratories, Inc. Hercules, California, USA), and the amplification conditions were as follows: initial denaturation at 94 °C for 4 min and 30 cycles at 94 °C for 30 s, 56.2 °C for 30 s and 72 °C for 30 s; finally, an extension at 72 °C for 10 min. PCR products were visualized on 1% agarose gel, using a TM-10E UV transilluminator (UVP Upland, CA 91786, USA).

### Disk diffusion test for cefoxitin

In a 13×100 mm test tube with 3 ml of sterile 0.85% saline, a bacterial suspension adjusted to the 0.5 McFarland standard was prepared using a DENSIMAT equipment (bioMérieux SA, Marcy-l'Étoile, France). A sterile rayon swab was then moistened with the suspension, and the inoculum was distributed homogeneously on a plate of Mueller–Hinton agar (OXOID Ltd, Basingstoke, Hampshire, England). A 30 µg cefoxitin disc (OXOID Ltd, Basingstoke, Hampshire, England) was placed, and the plate was incubated in an Isotemp 750D microbiological incubator (ThermoFisher Scientific, Waltham, Massachusetts, USA) at 35 °C for 24 h. After that time, the diameter of the inhibition halo was measured using a vernier device. A diameter of ≤24 mm was interpreted as MR [6].

### Oxacillin screen agar

Mueller–Hinton agar (OXOID Ltd, Basingstoke, Hampshire, England) plates containing 4% NaCl (Sigma-Aldrich, St. Louis, MO, USA) and 6 µg ml^−1^ of oxacillin (Sigma-Aldrich, St. Louis, MO, USA) were prepared. Plates were inoculated with 10 µl of 0.5 McFarland suspension of the isolate by streaking in one quadrant and incubated at 35 °C for 24 h. Plates were carefully observed under transmitted light; any growth after 24 h was considered oxacillin-resistant.

### Disk diffusion D-zone test

For the detection of inducible resistance to clindamycin, erythromycin (15 µg) and clindamycin (2 µg) discs (OXOID Ltd, Basingstoke, Hampshire, England) were placed on Mueller–Hinton agar (OXOID Ltd, Basingstoke, Hampshire, England) 15 mm apart. After a 24 h incubation at 35 °C, a positive test (*iMLS_B* phenotype) was defined as the flattening of the clindamycin inhibition zone in the area adjacent to the erythromycin disc, forming a ‘D’ shape. In contrast, the constitutive phenotype (*cMLS_B*) was identified in isolates showing resistance to both erythromycin and clindamycin with no inhibition zones around the discs. Finally, the *MS* phenotype was defined as resistance to erythromycin with a circular, unaffected zone of inhibition around the clindamycin disc (negative D-test).

### Statistical analysis

Data obtained from phenotypic susceptibility tests [oxacillin agar dilution (AD) and cefoxitin disc diffusion] and genotypic detection (*mecA*) were collected in a Microsoft Excel database and processed using the statistical software GraphPad Prism v5.0. Absolute frequencies and percentages were used to characterize *mecA* gene prevalence and resistance profiles in neonatal and adult populations. To determine the relationship between the presence of the gene and phenotypic resistance, Pearson’s Chi-square (*X^2^*) test was applied; when expected cell counts were <5, Fisher’s Exact Test was used to ensure statistical rigour. A value of *P*<0.05 was considered statistically significant. Sensitivity (S) and Specificity (SP) were calculated, using PCR results as the reference (‘gold’ standard). Confidence intervals were established at 95% (95% CI). The degree of agreement between genotype and phenotype was quantified using Cohen’s Kappa coefficient (*κ*) with 95% CI. *κ* values were interpreted under the categorical framework originally proposed by Landis and Koch and restated in McHugh, in which *κ*<0.00 indicates agreement worse than chance; 0.00–0.20, slight; 0.21–0.40, fair; 0.41–0.60 moderate; 0.61–0.80, substantial; and 0.81–1.00, almost perfect [25, 26].

## Results

### Population characterization and clinical origin

Over an 8-year period (March 2006 to November 2014), 664 strains of *Staphylococcus* were obtained from the INPer ‘Isidro Espinoza de los Reyes’, which is a tertiary level hospital where high-risk pregnant women, patients with gynaecological problems, premature newborns and gestational pregnancy loss are managed.

From the samples, 552 isolates (83.1%) were obtained from newborns, while 112 (16.9%) were from adult women. Regarding the level of care for newborns, most isolates (524; 94.9%) were obtained from those in the Intensive Care Unit (ICU), with the remaining 28 (5.1%) originating from the general inpatient wards. Of the 112 adult samples, 9 (8.0%) were from patients in the ICU, 87 (77.7%) from general inpatients and 16 (14.3%) from outpatients. The site of infection varied by patient age (Table 1). For neonates, most isolates were obtained from catheters (237; 42.9%) and blood (198; 35.8%). In contrast, adult isolates were primarily recovered from surgical wounds (23; 23.2%), blood (14; 13.4%) and urine (14; 13.4%) (Table 1).

**Table 1.** Comparison of disc diffusion test results of cefoxitin with detection of *mecA* gene in *S. epidermidis* and *S. hominis* isolates collected from different clinical samples in neonates (A) and adults (B)

A								
	*S. epidermidis*				*S. hominis*			
	(no. of isolate)*				(no. of isolate)*			
	mecA+		mecA-		mecA+		mecA-	
	CFX R	CFX S	CFX R	CFX S	CFX R	CFX S	CFX R	CFX S
Catheter	125	57	22	20	5	1	1	0
Blood	106	25	11	19	16	4	2	8
CSF	15	3	2	4	6	1	1	0
Conjunctival	14	11	1	3	1	1	1	0
Urine	11	4	2	7	0	0	0	0
Surgical wound	5	4	3	0	0	0	0	0
Bronchial aspirate	2	2	0	0	0	0	0	0
Pleural	0	0	2	0	0	0	0	0
Abscess	1	0	0	0	0	0	0	0
Cellulitis	1	0	0	0	0	0	0	0
Intraabdominal aspirate	1	0	0	0	0	0	0	0
Ascites fluid	0	0	0	1	0	0	0	0
Otic	0	1	0	0	0	0	0	0
Umbilical	0	1	0	0	0	0	0	0
n	281	108	43	54	28	7	5	8
	*n*=486				*n*=48			
B								
	***S. epidermidis***				***S. hominis***			
	**(no. of isolate)∗**				**(no. of isolate)∗**			
	***mecA+***		***mecA−***		***mecA+***		***mecA−***	
	**CFX R**	**CFX S**	**CFX R**	**CFX S**	**CFX R**	**CFX S**	**CFX R**	**CFX S**
Surgical wound	9	8	1	8	0	0	1	0
Blood	5	6	0	4	1	0	1	1
Catheter	3	3	0	3	1	0	0	1
Ascites fluid	3	2	1	1	0	0	0	0
Soft tissues	1	3	0	2	0	0	0	0
Peritoneal fluid	1	2	0	1	0	0	0	0
Urine	1	8	2	4	1	0	0	1
Cellulitis	1	0	0	1	0	0	0	1
Intraabdominal aspirate	1	0	0	1	0	0	0	0
Conjunctival	1	0	1	0	0	0	0	0
Abscess	0	1	0	1	0	0	0	0
Uterine cavity	0	1	0	0	0	0	0	0
Fistula	0	0	1	0	0	0	0	0
CSF	0	0	0	1	0	0	0	0
n	26	34	6	27	3	0	2	4
	*n*=93				*n*=9			

*∗mecA*−, *mecA* negative; *mecA+*, *mecA* positive; CFX R, cefoxitin resistant; CFX S, cefoxitin susceptible; CSF, Cerebrospinal fluid.

The 664 samples were analysed using the VITEK 2 system according to the manufacturer’s instructions. All isolates belonged to the *Staphylococcus* genus, with *S. epidermidis* being the most prevalent species (579 isolates; 87.2%). Of these, 93 (16.1%) were from adults and 486 (83.9%) were from newborns. The second most representative species was *S. hominis* with 57 isolates (8.6%), comprising 9 (15.8%) from adults and 48 (84.2%) from newborns. The remaining isolates consisted of other CoNS species. Further phenotypic validation of these isolates was conducted using phenotypic tests, including growth on mannitol salt agar and catalase and coagulase testing. Additionally, genomic DNA was extracted from the isolates for PCR analysis of the *coa* gene. Molecular analysis confirmed that 100% of the isolates were identified as CoNS, as evidenced by a negative result for the *coa* gene.

### Genotype–phenotype correlation for the detection of *mecA*

To identify the genetic determinants of MR, 579 *S*. *epidermidis* isolates and 57 *S*. *hominis* isolates were analysed for the presence of the *mecA* gene by PCR. In *S. epidermidis*, the *mecA* gene prevalence was 80% (*n*=389) in the neonatal population and 64.5% (*n*=60) in adults (Table 1). Validation of phenotypic susceptibility tests against the gold standard (PCR) demonstrated performance stratification depending on the test and population studied. Oxacillin [determined by MIC via AD at the CLSI breakpoint (≥0.5 mg l^−1^)] was the most sensitive marker in neonates (87.1%, 95% CI: 83.5–90.1%), surpassing cefoxitin (disc diffusion, ≤24 mm), which reached 72.2% (95% CI: 67.5–76.6%) in the same neonatal cohort (Table 2). However, both markers exhibited limited specificity (56.7% for oxacillin and 55.7% for cefoxitin), indicating that both phenotypic tests generate a substantial proportion of false-positive results within the analysed isolate cohort. Chi-square (*X^2^*) analysis confirmed that oxacillin is associated with the presence of the *mecA* gene in neonates (*X^2^*=88.13; *P*<0.001) and in adults (*X^2^*=28.53; *P*<0.001) (Table 3). Conversely, cefoxitin was also significantly associated with *mecA* in this same adult subgroup; the markedly lower *X^2^* (5.97, *P*=0.0145) than in the neonatal cohort (*X^2^*=27.21, *P*<0.001) reflects the reduced statistical power associated with the smaller sample size (*n*=93 versus *n*=486). Cohen’s kappa index (*κ*) further contextualized these observations: oxacillin showed moderate agreement with the PCR reference in both *S. epidermidis* subgroups (*κ*=0.44 in neonates and *κ*=0.54 in adults), whereas cefoxitin showed only fair agreement with the PCR reference in both *S. epidermidis* subgroups (*κ=*0.22 in neonates; *κ*=0.21 in adults), supporting the position that cefoxitin DD is unsuitable as a standalone screening marker for *mecA* in this setting (Table 3).

**Table 2.** Comparison of *mecA* gene detection versus oxacillin and cefoxitin disc diffusion susceptibility profiles in *S. epidermidis* and *S. hominis* isolates from neonates and adults.

		*mecA+* *(no. of isolates)^a^*		*mecA−* *(no. of isolates)^a^*		*mecA+* *(no. of isolates)^a^*		*mecA−* *(no. of isolates)^a^*	
**Sample**	**Species**	**CFX-DD resistant**	**CFX-DD** **susceptible**	**CFX-DD resistant**	**CFX-DD susceptible**	**OXA- resistant**	**OXA-** **susceptible**	**OXA- resistant**	**OXA-** **susceptible**
*Neonates*	*S. epidermidis*	281	108	43	54	339	50	42	55
*Adults*	*S. epidermidis*	26	34	6	27	47	13	7	26
*Neonates*	*S. hominis*	28	7	5	8	25	10	2	11
*Adults*	*S. hominis*	3	0	2	4	2	1	3	3

*mecA−, *mecA* negative; *mecA+*, mecA positive; CFX-DD, Disc diffusion cefoxitin; OXA, oxacillin

CFX DD, Cefoxitin disc diffusion; OXA, oxacillin.

**Table 3.** Sensitivity, specificity and Kappa index of phenotypic methods versus *mecA* PCR for MR identification in clinical isolates of *S. epidermidis* and *S. hominis* derived from neonatal and adult patient populations*

Species	Population	Antibiotic	χ2	***P*-value**	S (%)	SP (%)	*κ*
*S. epidermidis*	Neonates	OXA	88.13	<0.0001	87.15	56.7	0.44
		CFX	27.21	<0.0001	72.2	55.7	0.22
	Adults	OXA	28.53	<0.0001	78.3	78.8	0.54
		CFX	5.97	0.0145	43.3	81.8	0.21
*S. hominis*	Neonates	OXA	7.11	0.0077	71.4	84.6	0.48
		CFX	6.67	0.0098	80.0	61.5	0.39
	Adults	OXA	0.225	0.6353†	66.7	50.0	0.14
		CFX	3.6	0.119†	100	66.7	0.60

* CFX, cefoxitin; OXA, oxacillin; X2, chi-square; S, sensitivity; SP, specificity; κ, Kappa index.

†Fisher test.

For *S. hominis*, 57 isolates were analysed, consisting of 48 (84.2%) from neonates and 9 (15.8%) from adults. The *mecA* gene was detected in 72.9% (*n*=35) in the neonatal group and 33.3% (*n*=3) in the adult population (Table 2). Phenotypic marker performance showed inconsistent diagnostic behaviour, differing markedly from the patterns observed in *S. epidermidis*. Among neonates (*n*=48), cefoxitin demonstrated higher sensitivity than oxacillin (80 versus 71.4%), although specificity for both markers remained limited (61.5% for cefoxitin and 84.6% for oxacillin), reflecting the small *mecA* negative denominator (*n*=13) in this subgroup. These specificity values, together with the *κ* findings reported, point to high rates of phenotypic-method disagreement in *S. hominis* neonates and limit the operational value of either method as a standalone screening modality in this species (Table 3). In the adult subgroup (*n*=9), the small sample size prevented precise estimation: cefoxitin exhibited 100% sensitivity but 66.7% specificity, suggesting a tendency toward overestimation of MR from disc diffusion results in this dataset.

Due to the distribution of the data, Fisher’s exact test was applied, revealing only a weak statistical association. In neonates, both phenotypic markers reached strong statistical association with the *mecA* gene by Fisher’s exact test: oxacillin (*P*=0.0077) and cefoxitin (*P*=0.0098), both values falling below the *P*<0.05 threshold defined a priori. In the adult population, the limited number of positive cases meant that Fisher’s exact test failed to yield statistical significance for either phenotypic test (cefoxitin *P*=0.119; oxacillin *P*=0.6353).

Concordance assessments further underscored the unreliability of conventional phenotypic methods for *S. hominis*. In neonates, *κ* values classified as fair agreement for cefoxitin (*κ*=0.39) and moderate agreement for oxacillin (*κ*=0.48), with the higher *κ* for oxacillin reflecting its superior specificity (84.6%) despite its lower sensitivity (71.4%). In the adult subgroup (*n*=9; 3 *mecA* positive), the small sample size prevented reliable concordance estimation; oxacillin returned *κ*=0.14 (slight agreement) and cefoxitin returned *κ*=0.60 (moderate agreement). These estimates, particularly those derived from the nine-isolate adult subgroup, preclude extrapolation to species-level evidence and reflect the limitations of phenotypic reliability at the current sample size.

### Multi-resistance profile and macrolide and lincosamide resistance phenotypes

For the characterization of the macrolide–lincosamide resistance landscape within the CoNS collection, two complementary dimensions were investigated: the rates of erythromycin and clindamycin resistance stratified by *mecA* status, and the distribution of inducible, constitutive and MS phenotypes among isolates demonstrating resistance to either or both markers. The first dimension is summarized graphically in Fig. 1 and reported in full, with the row-level counts and percentages for each strata, in Table S1; the second is detailed in Table 4.

**Fig. 1.**
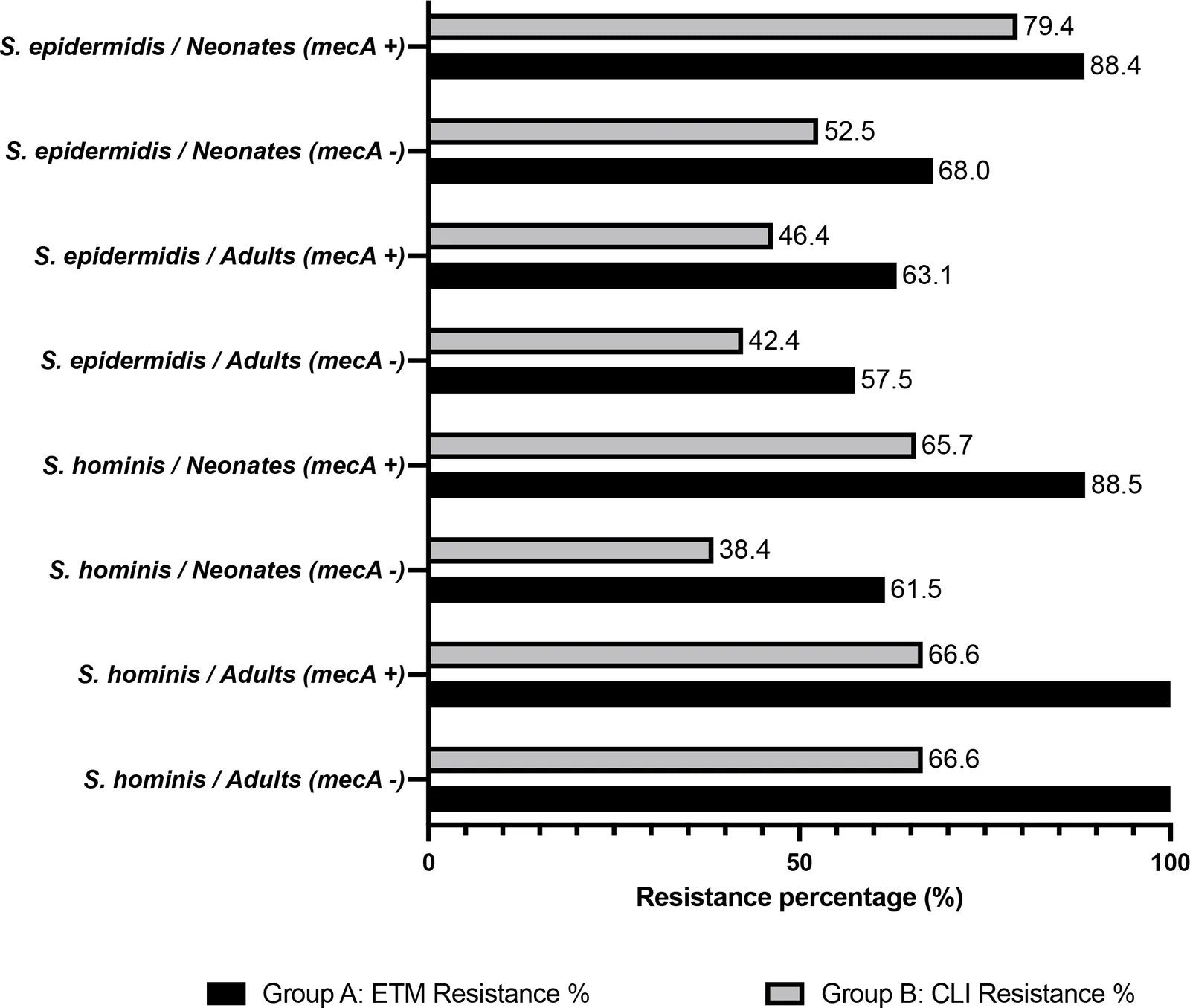
Macrolide and lincosamide resistance rates by *mecA* status, species and cohort. ^a^ETM, erythromycin; CLI, clindamycin.

**Table 4.** Phenotypic characterization of the MLS_B mechanism (D-test) and its correlation with the *mecA* gene in *S. epidermidis* and *S. hominis* isolates

Species	Patient cohort	n	Inducible (*iMLS_B_*) n (%)	Constitutive (*cMLS_B_*) n (%)	MS phenotype n (%)	Total macrolide resistance (%)*
*S. epidermidis*	Neonates	486	5 (1.0)	358 (73.7)	47 (9.7)	84.4
	Adults	93	1 (1.1)	40 (43.0)	16 (17.2)	61.3
*S. hominis*	Neonates	48	10 (20.8)	24 (50)	3 (6.2)	77.0
	Adults	9	2 (22.2)	6 (66.7)	1 (11.1)	100

*iMLS_B_*, inducible clindamycin resistance phenotype (positive D-test); *cMLS_B,_* Constitutive resistance phenotype (negative D-test with initial resistance to both erythromycin and clindamycin); MS phenotype, macrolide–streptogramin resistance phenotype (negative D-test with resistance to erythromycin but susceptibility to clindamycin); n(%), percentages were calculated based on the total number of isolates per species and population group.

In *S. epidermidis*, examination of the *mecA*-stratified resistance landscape revealed that erythromycin resistance was elevated across all strata, peaking at 88.4% in *mecA+* neonates and declining stepwise through *mecA−* neonates (68.0%), *mecA+* adults (63.1%) and *mecA−* adults (57.5%), exceeding 75% only in the *mecA+* neonatal subgroup. Clindamycin resistance tracked more closely with *mecA* carriage: in the neonatal cohort, clindamycin resistance reached 79.4% in *mecA*+ isolates but only 52.6% in *mecA*− isolates, a 26.8-percentage-point gap consistent with the persistence of *erm*-mediated resistance in multidrug-selected Neonatal Intensive Care Unit (NICU) clones. In adults, both *mecA* strata returned lower and more convergent clindamycin resistance values (48.3% in *mecA*+; 42.4% in *mecA*−), consistent with reduced selective pressure outside the NICU setting (Fig. 1) .

In *S. hominis*, a departure from the *S. epidermidis* pattern is observed in the same Fig. 1 and Table S1 comparison. Clindamycin resistance remained elevated in *mecA*+ neonates (65.7%) but dropped sharply in *mecA*− neonates (38.4%), an inversion of the gradient observed in *S. epidermidis*, where clindamycin resistance was higher in *mecA+*+ isolates and consistent with the broader observation that *S. hominis* carries clindamycin-resistance determinants independently of the *SCCmec* cassette. Among the small adult subgroup (*n*=9), clindamycin resistance was elevated in both *mecA* strata (66.7% in each); since the adult subgroup comprises only three *mecA+*+ and six *mecA−* isolates, these percentages should be interpreted as point estimates rather than species-level evidence.

A complementary phenotype distribution was observed. In neonatal *S. epidermidis*, the constitutive phenotype (cMLS_B) dominated, affecting 73.7% (*n*=358) of isolates; the inducible phenotype (iMLS_B) was rare at 1.0% (*n*=5) and the macrolide-susceptible MS phenotype accounted for 9.7% (*n*=47). Among adults, cMLS_B remained the most frequent pattern but at the lower rate of 43.0% (*n*=40); the MS phenotype was relatively more frequent in adults (17.2%, *n*=16) than in neonates (9.7%, *n*=47), mirroring the lower overall multidrug-resistance burden in non-ICU settings.

In *S. hominis*, the phenotype distribution was distinctive in two respects. The inducible phenotype (*iMLS_B*) was substantially more frequent in *S. hominis* than in *S. epidermidis* across both cohorts (20.8%, *n*=10 in neonates; 22.2%, *n*=2 in adults). *When iMLS_B* and *cMLS_B* were combined, 70.8% (34 of 48) of neonatal *S. hominis* isolates carried a clindamycin-resistance mechanism, 20.8% *iMLS_B* (*n*=10) plus 50% *cMLS_B* (*n*=24), that could compromise empirical clindamycin therapy. This rate is comparable to 74.7% seen in neonatal *S. epidermidis* (*n*=363 of 486 isolates; 1.0% *iMLS_B*, *n*=5, plus 73.7% *cMLS_B*, *n*=358), but with a markedly higher contribution from the inducible phenotype in *S. hominis* (20.8%, *n*=10) than in *S. epidermidis* (1.0%, *n*=5).

In conjunction, Fig. 1, Tables 4 and S1 delineate a two-axis resistance landscape wherein (i) phenotypic resistance rates and the *mecA* genotype do not coincide uniformly across species and cohorts; (ii) inducible clindamycin resistance, remaining mechanistically obscured by conventional antibiograms, is concentrated in *S. hominis* and concentrated further in the NICU setting; and (iii) the high cumulative clindamycin-therapy-invalidation rate in neonatal *S. hominis* (70.8%) constitutes the operational justification for systematic D-test screening of erythromycin-resistant CoNS isolates in this population.

## Discussion

While oxacillin AD, cefoxitin disc diffusion and *mecA* PCR are long-established techniques, well documented in reference-centre settings, phenotypic susceptibility testing remains the operational standard in Mexican governmental hospitals, as the requisite technical infrastructure (e.g. equipment and expertise) for routine molecular confirmation is not uniformly available at the secondary-care level [27]. Under these conditions, the application of validated breakpoints to a Mexican public-sector CoNS population has not previously been combined with species-stratified analysis across *S. epidermidis* and *S. hominis* together with formal phenotype–genotype concordance analysis on both a neonatal and an adult cohort. The closest Mexican precedent combined cefoxitin disc testing with mecA PCR in a tertiary-care setting under a hospital-surveillance design [28] and, more recently, on a smaller mixed-species collection [29]; neither analysed Cohen’s *κ* concordance, neither separated neonates from adults and neither stratified by CoNS species. The present study contributes, therefore, a region-specific, species-stratified validation of an established method-pair in a fixed-resource public-sector setting.

CoNS, historically considered contaminants of clinical specimens, have emerged in recent decades as the main opportunistic pathogens in the modern hospital environment [30]. This paradigm shift is primarily attributed to the increased utilization of invasive medical devices and the biofilm-forming capabilities of these species, which confer protection against host immune responses and antimicrobial agents [3]. Currently, CoNS represent the most frequent cause of bloodstream infections and late-onset sepsis in vulnerable populations, especially in preterm infants and immunocompromised patients [31].

Driven by this global epidemiological shift towards opportunistic hospital-acquired infections, NICUs function as unique ecological niches [32]. The prevalence of the *mecA* gene in neonates was determined to be 80% in *S. epidermidis* and 72.9% in *S. hominis* (35/48 isolates), reflecting the dissemination of multidrug-resistant endemic clones adapted to the selective pressure of the hospital environment [30, 32]. The clinical relevance of these isolates is supported by their anatomical origin: among neonates, 46.1% of *S. epidermidis* were recovered from catheters and 62.5% of *S. hominis* from blood, with a worrying 16.7% in CSF. These findings confirm the role of these isolates as true pathogens in severe invasive infections indicative of invasive pathogenic potential rather than commensal skin colonization. In contrast, the adult population exhibited greater diversity in the origin of the samples, with a significant presence in surgical wounds (28%) and urine (16.1%). This heterogeneity suggests a distinct epidemiology, where isolates are exposed to intermittent antibiotic pressures [33, 34]. This variability in sample origin is consistent with the observation that statistical agreement was robust in neonates but null in specific adult patient subgroups, supporting the interpretation that the clinical environment directly influences the stability of resistance-genotype expression at the phenotypic level. This conceptual framing aligns with the characterization of NICUs as continuous microbial reservoirs in which environmental and infant-gut microbiomes overlap [35], and it is reinforced by recent evidence of a high multidrug-resistance burden in *S. hominis* collections, which positions *S. hominis* as a clinically significant opportunistic reservoir in its own right [36]

Cefoxitin is a substantially more efficient *mecR1* inducer than oxacillin, which theoretically favours detection of MR by forcing expression of the *mecA* gene. Within the collection, however, this theoretical advantage did not translate into uniformly higher phenotypic agreement with the PCR. In *S. epidermidis* oxacillin by AD with 4% NaCl and 24 h of incubation returned higher *κ* than cefoxitin across both neonatal (*κ*=0.44 versus *κ*=0.22) and adult (*κ*=0.54 versus *κ*=0.21); all values fell within the ‘moderate’ (*κ*=0.41–0.60) or ‘fair’ (*κ*=0.21–0.40) categories of the Landis and Koch scale [25, 26]. Although McHugh explicitly notes that ‘moderate’ agreement is enough for clinical concordance, it is not a regulatory-grade basis for substituting one phenotypic method for another. The higher *κ* observed for oxacillin is therefore best interpreted as a gradient of concordance rather than as a generalizable clinical recommendation. This pattern aligns with data from prior reports suggesting that standardized 4% NaCl/24 h incubation conditions optimize the detection of PBP2a-mediated resistance [6].

For *S. hominis*, the comparison was constrained by an unequally distributed cohort (*n*=48 neonates and *n*=9 adults). In neonates, the two phenotypic markers showed complementary error profiles: cefoxitin had high sensitivity (80%) but limited specificity (61.50%), whereas oxacillin showed limited sensitivity (71.4%) but appreciably higher specificity (84.6%). Under the Landis and Koch scale, the *κ* values for neonatal *S. hominis* classified agreement as fair for cefoxitin (*κ*=0.39) and moderate for oxacillin (*κ*=0.48), with the higher *κ* for oxacillin reflecting its superior specificity (84.6%) despite its lower sensitivity (71.4%). In the small adult subgroup (*n*=9), oxacillin yielded *κ*=0.14, corresponding to slight agreement under the scale, whereas cefoxitin yielded *κ*=0.60, which on the Landis and Koch scale is classifiable as moderate agreement. However, given the inclusion of only nine isolates, this point estimate is not interpreted as a robust species-level claim, consistent with McHugh’s caution that diagnostic inferences should not rest on a single *κ* value derived from limited sample sizes.

The *χ*² or Fisher’s exact tests reported determined whether each phenotypic marker was individually associated with the *mecA* gene, not whether the two markers differed in sensitivity; a formal paired sensitivity comparison between the two phenotypic methods would require McNemar’s test on discordant pairs, which was not performed. Compared with regional CoNS surveys in which phenotypic tests were evaluated without *κ* concordance and without NICU–adult stratification [28, 37], the present dataset provides a species-resolved, age-stratified concordance analysis on a fixed-resource public-sector collection. The discrepancy between these species-specific findings and the more favourable cefoxitin performance reported in some Latin American surveys may plausibly reflect the inclusion in our collection of CoNS isolates with very low or heterogeneous levels of PBP2a expression, in which the standard cefoxitin 30 µg disc and the 24 mm zone-diameter breakpoint may be insufficient to push the measured zone below the resistance threshold. The observation that phenotypic detection findings for *S. epidermidis* show higher *κ* agreement for oxacillin than for cefoxitin sits alongside the broader observation from Fariña *et al*. in a regional cohort that biofilm production is more frequent in methicillin-resistant isolates, with both findings underscoring the clinical importance of identifying *mecA* carriage as accurately as possible in any CoNS isolate [38].

When erythromycin resistance rates are stratified by *mecA* status, species and cohort, the resistance landscape remains elevated across all categories, with the highest macrolide resistance in neonatal *S. epidermidis* (84.4%; Table 4). The *cMLS_B* phenotype alone reached 73.7% in neonatal *S. epidermidis*, exceeding the 46% reported by Gatermann *et al*. in 2007 and aligning with the upward trend documented by Szczuka *et al*. in clinical *S. hominis* isolates; these figures confirm a sustained global increase in macrolide resistance over the past two decades [39, 40].

Regarding clindamycin, a divergent resistance landscape between species is observed. In *S. hominis*, the 20.8% prevalence of the inducible phenotype (*iMLS_B*) underscores a silent clinical risk: an antibiogram that reports clindamycin as susceptible on an erythromycin-resistant *S. hominis* isolate may produce therapeutic failure. In contrast, *S. epidermidis* showed an overwhelming predominance of the constitutive mechanism (*cMLS_B*) at 73.7% in neonates, compared to only 43.0% in adults. The ~30-percentage-point gradient is consistent with the hypothesis of stronger selective pressure for multidrug-resistant clones in NICU settings relative to general wards. Interestingly, the *MS* phenotype was more prevalent in the adult population (17.2%) than in neonates (9.7%). The 17.2% versus 9.7% difference is consistent in direction with the hypothesis that in non-ICU environments, resistance may be mediated more frequently by *msr*-type efflux pumps, although this inference requires further molecular confirmation (Fig. 1).

The therapeutic complexity is compounded by the observation that *cMLS_B* and *iMLS_B* phenotypes were detected in some *mecA*negative isolates. The apparent ‘independence’ documented is a descriptive case-level observation rather than a tested population-level association. Mechanistically, the co-occurrence of *mecA* carriage and erythromycin/clindamycin resistance in NICU isolates without physical co-carriage on a single cassette is expected: erythromycin and clindamycin resistance in staphylococci is encoded by *ermA, ermB* and *ermC*, which are typically carried on transposons such as Tn551*/*Tn917 or on small plasmids rather than within the SCCmec element itself [41]. The NICU co-resistance profile is therefore consistent with co-selection under sustained multi-class antibiotic pressure rather than with any direct genetic-linkage mechanism, and the observation reinforces the role of the NICU as a high-intensity reservoir for combined resistance.

Detection in *S. hominis* neonates, of 20.8% inducible clindamycin resistance (*iMLS_B*), coupled with 50.0% constitutive resistance (*cMLS_B*), implying that 70.8% of isolates carry a mechanism that would invalidate clindamycin therapy regardless of any susceptibility reported by conventional antibiogram (Table 4; Fig. 1). Combined with the small adult subgroup (*n*=9) showing 22.2% (*n*=2) *iMLS_B* and 11.1% (*n*=1) *MS*, these values should be interpreted with substantial caution given the very small sample size. The data position *S. hominis* as a particularly unpredictable resistance reservoir in the NICU setting and support the practical recommendation that clindamycin should not be prescribed empirically in this species without prior D-test screening on erythromycin-resistant isolates or, where available, PCR confirmation of *ermA/B/C* carriage.

Despite the methodological soundness and large size of the neonatal sample, limitations inherent to the study design must be noted. First, the numerical disparity between the neonatal and adult populations analysed (*N*=534 versus *N*=102, comprising 486 versus 93 *S*. *epidermidis* and 48 versus 9 *S*. *hominis*) limited the statistical power in the latter subgroup, as reflected in the elevated χ² *P*-values and the weak *κ* indices observed in some adult subgroups with limited sample sizes (e.g. in *S. hominis* isolates from adults, the χ²/Fisher test for cefoxitin returned *P*=0.119 with *κ*=0.60, and for oxacillin *P*=0.6353 with *κ*=0.14). Second, the absence of molecular typing by pulsed-field gel electrophoresis or whole-genome sequencing (WGS) prevented confirmation of the clonal structure suggested by the multi-resistance profiles; the co-resistance profiles observed cannot be interpreted as evidence of physical co-carriage of resistance genes on a single cassette, and WGS data would be required to resolve this structural question. This consideration reinforces the corollary that, because *ermA, ermB* and *ermC* genes are typically carried on transposons such as Tn551/Tn917 or on small plasmids rather than within the *SCCmec* element itself, phenotypic co-resistance in NICU isolates is most plausibly explained by co-selection under sustained antibiotic pressure rather than by genetic linkage within a single cassette. Focusing exclusively on the *mecA* gene also left other less frequent determinants, such as *mecC* or mutations in native PBPs, out of analysis. Finally, the single-centre nature of the study implies that the results reflect selective pressure and local antimicrobial use policies; therefore, extrapolation of these findings to other hospitals should be carried out with caution.

Operationally, the management of CoNS infections in the NICU requires a multifaceted approach. Although oxacillin, when assessed by the reference method, proved to be an acceptable phenotypic predictor in the study cohort, the invasive-disease profile of *S. hominis* in neonates, with 62.5% of isolates recovered from blood and 16.7% from CSF, together with the 20.8% *iMLS_B* prevalence in neonatal *S. hominis* isolates cited above, makes systematic D-test screening of erythromycin-resistant isolates and, where available, *mecA* PCR confirmation necessary in this group [40]; the integration of sample-origin information (catheter, blood, CSF), D-test screening on every erythromycin-resistant CoNS isolate and molecular confirmation where available can support safe antimicrobial therapy in the neonatal population [42].

Data presented herein demonstrate that oxacillin AD maintains clinically usable diagnostic validity as a frontline phenotypic test in the NICU setting; at the same time, the inducibility of *mecA* expression by the *mecR1/mecI* regulatory system, combined with the documented diversity of CoNS species, suggests that no single phenotypic method can be relied upon across the full neonatal CoNS population, making species-stratified surveillance a suitable response to the continuing evolution of multiresistance phenotypes [43].

## Supplementary material

10.1099/jmm.0.002207 Table S1.

## Abbreviations

AD: agar dilution
BHI: brain heart infusion
BSI: bloodstream infection
CFX: cefoxitin
CI: confidence interval
CLI: clindamycin
CLSI: Clinical and Laboratory Standards Institute
cMLS_B: constitutive macrolide-lincosamide-streptogramin B resistance
CoNS: coagulase-negative staphylococci
CSF: cerebrospinal fluid
DD: disk diffusion
DD: Disc Diffusion
ETM: erythromycin
HAI: healthcare-associated infection
ICU: intensive care unit
iMLS_B: inducible macrolide-lincosamide-streptogramin B resistance
INPer: National Institute of Perinatology
MGE: mobile genetic element
MIC: minimum inhibitory concentration
MR: methicillin resistance
MS: macrolide-streptogramin phenotype
MSA: mannitol salt agar
NICU: neonatal intensive care unit
OXA: oxacillin
PBP: penicillin-binding protein
PBP2a: penicillin-binding protein 2a
SCCmec: staphylococcal cassette chromosome mec
WGS: whole-genome sequencing
