# Supplementary material for: Correlation between phenotypic disc diffusion test and PCR molecular test for detection of methicillin resistance in Staphylococcus epidermidis and Staphylococcus hominis
Source: J Med Microbiol. 2026 Nov 3;75(11):002207. doi: 10.1099/jmm.0.002207 (PMC13730459; doi:10.1099/jmm.0.002207)
Supplement: Supplementary file 1 [file jmm-75-02207-s001.pdf]

12. Supplementary Tables

Supplementary Table 1. Prevalence of phenotypic resistance to Erythromycin and Clindamycin in *S. epidermidis* and *S. hominis* in neonatal and adult populations.

**Species**	**Population**	***mecA* status**	**ETM (R)**	**ETM (I)**	**ETM (S)**	**ETM Resistance %**	**CLI (R)**	**CLI (S)**	**CLI Resistance %**
S. epidermidis	Neonates	*mecA* (+)	344	6	39	**88.40%**	309	80	**79.43%**
		mecA (-)	66	3	28	68.00%	51	46	52.58%
	Adults	*mecA* (+)	38	4	18	**63.10%**	29	31	48.33%
		mecA (-)	19	3	11	57.50%	14	19	42.43%
S. hominis	Neonates	*mecA* (+)	31	1	3	**88.50%**	23	12	**65.71%**
		mecA (-)	8	0	5	61.50%	5	8	38.46%
	Adults	*mecA* (+)	3	0	0	**100%**	2	1	**66.67%**
		mecA (-)	6	0	0	**100%**	4	2	**66.67%**

[a] ETM, erythromycin; CLI, clindamycin; R, resistant; I, intermediate; S, susceptible
